# Simplified panicle fertilization is applicable to *japonica* cultivars, but splits are preferred in *indica* rice for a higher paddy yield under wheat straw return

**DOI:** 10.3389/fpls.2024.1273774

**Published:** 2024-01-30

**Authors:** Xiaowei Shu, Xiaoxiang Zhang, Shushen Wang, Tong Fu, Zhouyu Ding, Ying Yang, Zihan Wang, Shiru Zhao, Jiejiao Xu, Juan Zhou, Jing Ju, Jianye Huang, Youli Yao, Yulong Wang, Guichun Dong

**Affiliations:** ^1^ Jiangsu Key Laboratory of Crop Cultivation and Physiology/Co-Innovation Center for Modern Production Technology of Grain Crops, Yangzhou University, Yangzhou, Jiangsu, China; ^2^ Lixiahe Agricultural Research Institute of Jiangsu Province, Jiangsu Key Laboratory of Crop Genomics and Molecular Breeding, Yangzhou, Jiangsu, China; ^3^ College of Environmental Science and Engineering, Yangzhou University, Yangzhou, Jiangsu, China

**Keywords:** wheat straw return, panicle fertilization, spikelet differentiation and degeneration, yield, *indica*, *japonica*

## Abstract

**Introduction:**

The panicle fertilization strategy for japonica and indica rice under wheat straw return (SR) has not been updated, especially on the elaboration of their impacts on spikelet differentiation and degeneration. This study aimed to verify the hypothesis that SR increases spikelet number by reducing spikelet degeneration and to explore the possibility of simplifying panicle fertilization.

**Methods:**

In three consecutive years, four varieties of japonica and indica rice were field-grown in Yangzhou, Jiangsu Province, China. Six panicle fertilization rates and split treatments were applied to SR and no straw return (NR) conditions.

**Results:**

The results showed that SR promoted rice yield significantly by 3.77%, and the highest yields were obtained under the T2 (split panicle fertilization at the panicle initiation (PI) and spikelet primordium differentiation (SPD) stages) and T1 (panicle fertilization only at the PI stage) treatments, for indica and japonica rice, respectively. Correlation and path analysis revealed that the number of spikelets per panicle was the most attributable to yield variation. SR significantly increased the concentration of alkali hydrolyzable N in the soil 40 days after rice transplantation, significantly increased the nitrogen accumulation per stem (NA) during the SPD-pollen mother cell meiosis (PMC) stage, and increased the brassinosteroids level in the young panicles at the PMC stage. SR also reduced the degeneration rate of spikelets (DRS) and increased the number of surviving spikelets (NSS). The dry matter accumulation per stem was more important to increasing the NA in japonica rice at the PMC stage, whereas NA was more affected by the N content than the dry matter accumulation in indica rice. In japonica rice, panicle N application once only at the PI stage combined with the N released from SR was enough to improve the plant N content, reduce the DRS, and increase the NSS. For indica rice, split application of N panicle fertilization at both the PI and SPD stages was still necessary to achieve a maximum NSS.

**Discussion:**

In conclusion, under wheat SR practice, panicle fertilization could be simplified to once in japonica rice with a significant yield increase, whereas equal splits might still be optimal for indica rice.

## Introduction

Rice is a staple food for more than 65% of China’s population, making China both the largest producer and consumer of rice in the world (Huang et al., 2021). However, rice acreage in China has stagnated since the beginning of the twenty-first century, and the yield has reached a plateau as well (Cui et al., 2020). Before new high-yielding varieties are developed and adopted (Cheng et al., 2007), optimizing fertilizer input and increasing fertilizer efficiency are long-term strategies to sustain rice production in China without compromising yield (Peng et al., 2009; Chen et al., 2011). Nitrogen (N) fertilizer is crucial for maintaining and enhancing rice yield, but it is usually applied in multiple splits as basal, tiller, and panicle fertilizers during a growth season (Liu et al., 2020; Wei et al., 2021), which increases labor input and machinery operation costs and incites problems such as low N fertilizer use efficiency and high N loss from rice fields (Vitousek et al., 2009).

Rice yield can be improved by increasing the number of spikelets per panicle (Zhang et al., 2020; Liu et al., 2022), a critical yield component that is influenced by several factors, such as fertilizer management, temperature, moisture, light, and endogenous hormones (Wang et al., 2018). Among these factors, the application of panicle fertilizer is an effective manipulation of spikelet differentiation and degeneration (Kamiji et al., 2011). In the range of 0–216 kg N ha^−1^, increasing panicle N fertilization enhances spikelet differentiation, with spikelet degeneration tending to decrease (Li et al., 2023). Nitrogen application at the panicle initiation (PI) and spikelet primordium differentiation (SPD) stages increases the levels of brassinosteroids (BRs), such as 24-epicastasterone and 28-homobrassinolide in young rice panicles at the SPD and pollen mother cell meiosis (PMC) stages, respectively, leading to more spikelet per panicle thanks to more differentiated spikelets and less degenerated spikelets (Zhang et al., 2019).

Wheat straw is an important biomass resource, and wheat straw return (SR) to the field also increases the spikelet number in rice (Zhang et al., 2021; Zhang et al., 2022a; Li et al., 2023). Following SR, due to the initial high C/N ratio of the straw, microorganisms use N from the soil to decompose the straw, thereby generating a “microbial N fixation effect”, which reduces the overall N content of the soil (Cheshire et al., 1999; Zhao et al., 2016; Kalkhajeh et al., 2021; Liu et al., 2021; Fan et al., 2022). As straw-source carbon availability declines, microbes die in large numbers, releasing the N they fixed, and the soil N content increases (Zhao et al., 2016; Cao et al., 2018). The drop and rise of soil nutrient (especially N) content coincide with the tillering and panicle development periods of rice, respectively, which may reduce the tillering number per unit area and increase the spikelet number per panicle (Li et al., 2021; Zhang et al., 2021). Previous studies on the effect of SR on rice spikelets focused mainly on the number of surviving spikelets, which is the difference in the numbers of differentiated and degenerated spikelets. However, few studies have explored the changes of differentiated and degenerated spikelets under SR conditions.

In Chinese rice production, panicle fertilizer is usually applied in two splits, one at the PI stage and the other at the SPD stage (Wu et al., 2022). Jiangsu Province is a prominent rice-producing region in the middle and lower reaches of the Yangtze River in China, currently the country’s largest and most concentrated area under rice–wheat rotation, with abundant straw resources to return (Li et al., 2017). Over the past 20 years of implementation of SR practices in Jiangsu Province, rice producers and researchers have followed the traditional two-split panicle fertilization regime, irrespective of the N application rate (Chen et al., 2011; Yang et al., 2022). However, little is known about whether SR may alter panicle fertilization scenarios for rice plants. Fertilization practice may be simplified at a reduced frequency if the N released from SR is enough to pace up the panicle development need.

The objective of this research was to investigate how and why simplified panicle fertilization after SR promotes the yield formation of *indica* and *japonica* rice in Yangzhou, Jiangsu Province, China, including: (1) to determine the effect of SR on spikelet differentiation and degeneration in rice; (2) to analyze the reasons for differential responses to simplified panicle fertilization in *indica* and *japonica* rice after SR. Our field study provided evidence and a theoretical basis for developing a simplified panicle fertilization strategy for rice under SR practices.

## Materials and methods

### Description of the experimental site

**Figure 1:**
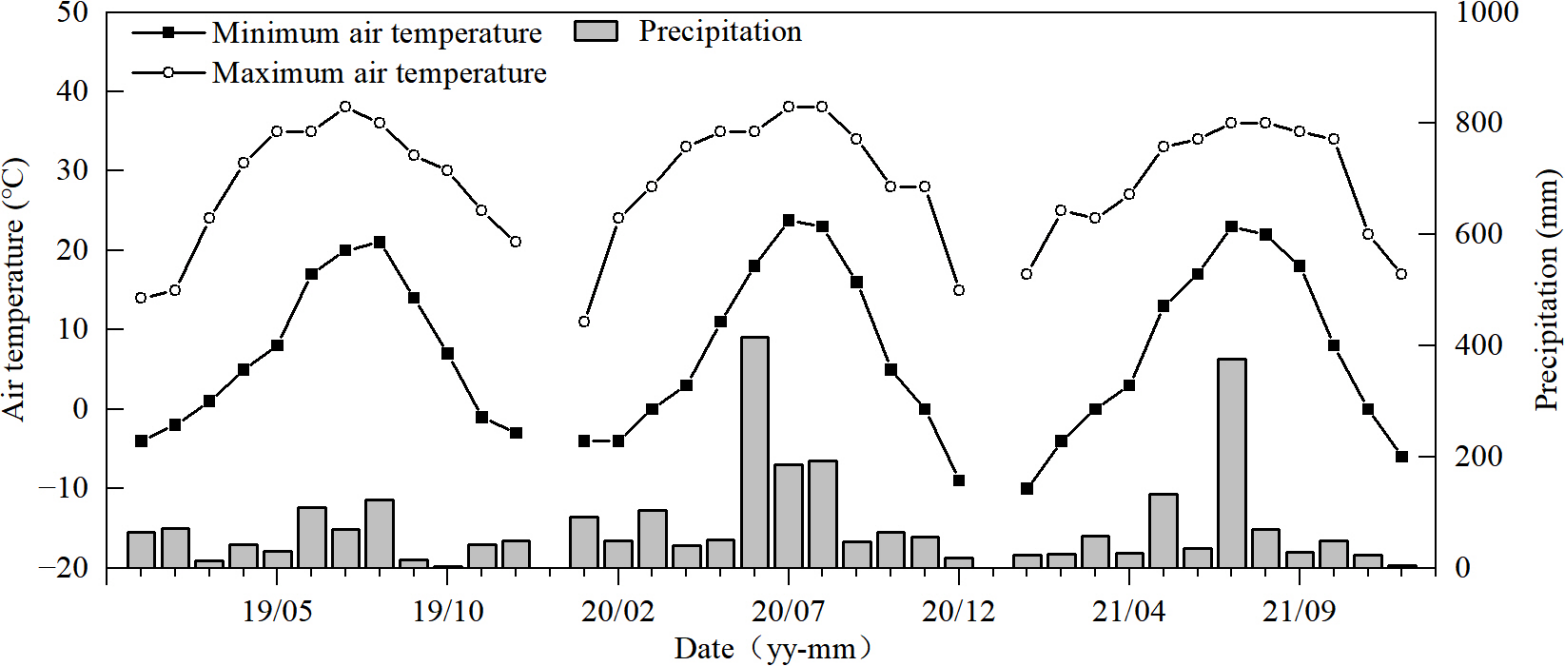
Temperature and precipitation during the rice growth seasons (2019–2021) in Yangzhou (Jiangsu Province, China).

The experiments were conducted in the 2019, 2020, and 2021 seasons at the experimental farm of the College of Agriculture at Yangzhou University, in Yangzhou (119°25′53″N, 32°23′43″E) of Jiangsu Province, China, located within a subtropical humid monsoon climate zone. The weather conditions are an annual mean temperature of 14.8°C, sunshine hours at 2,172.3, and precipitation at 1,049.4 mm (**Figure 1**). The soil in the experimental field is composed of sandy loam with 22.5 g kg^−1^ of organic matter, 1.74 g kg^−1^ of total nitrogen, 83.74 mg kg^−1^ of available nitrogen, 20.73 mg kg^−1^ of available phosphorus, and 92.52 mg kg^−1^ of available potassium.

### Crop culture and management

Four rice (*Oryza sativa* L.) varieties, namely, Nanjing 9108 and Wuyujing 3 (*japonica* rice) and Yangdao 6 and Yangliangyou 6 (*indica* rice), all released and commonly cultivated in Jiangsu Province, were employed in the trials. Rice seeds were acquired from Jiangsu King-earth Seed Limited Company (Yangzhou, Jiangsu, China) and directly sown on seedling trays measuring 28 cm × 58 cm. The nursery soil was mixed with common paddy field soil and a seedling enhancer (1:200, w/w) provided by Lixiahe District Agricultural Science Institute in Yangzhou, Jiangsu Province, China. The enhancer contains a mixture of plant-based enzymes, organic matter, humic acid, and plant-available N, phosphorus, and potassium, as well as antibiotics.

The arrangement of experimental plots was randomized within a block, and plots were separated by ridges embedded with plastic film for independent irrigation management to prevent carry-over of fertilizers. Each plot was 20 m^2^ (4 m × 5 m), with three replications for a treatment. Four seedlings per hill were transplanted for *japonica* cultivars and two for *indica* cultivars. After transplanting, the plants were irrigated to a depth of about 3 cm until the end of the tillering stage. The water was then drained, for about a week, to control the nonproductive tillers. About 3–5 cm of water was kept at subsequent stages until 7 days before harvest. Throughout the growth season, any pests, diseases, and weeds were controlled according to local practices to ensure the normal growth of rice plants (Dong et al., 2022).

### Experiment design

After the wheat (*Triticum aestivum* L., cv: Yangmai 25) harvest, the wheat straw (N content: 0.82%) was machine chopped to a length of 5–10 cm and plowed back into the field at an SR rate of 6 t ha^−1^. SR, or no straw return (NR), was set up as a split-plot major factor, and the subplot factors were rice variety and panicle fertilization treatment. Nitrogen fertilizer was applied at a rate of 270 kg N ha^−1^ with four splits at a ratio of 3.5:3:1.75:1.75 on the day before transplanting, mid-tillering (7 days after transplanting), PI, and SPD stages, respectively. Four panicle fertilization treatments were implemented, namely no panicle fertilization (CK), panicle fertilization at the PI stage (T1), at the PI and SPD stages (T2), and at the SPD stage (T3). In 2020 and 2021, two additional panicle fertilization treatments were tested (**Table 1**) to clarify the effects of the rate and ratio of panicle fertilization. The basal fertilizer was applied 1 day before transplanting, including P_2_O_5_ at 40 kg ha^−1^ and K_2_O at 100 kg ha^−1^.

**Table 1:** Setup of panicle fertilization in the experiments.

Year	Panicle fertilization treatment		N fertilization level (kg ha^−1^)	Base fertilizer (kg ha^−1^)	Tillering fertilizer (kg ha^−1^)	Panicle fertilizer (kg ha^−1^)	
						PI	SPD
2019−2021	CK		175.5	94.5	81	0	0
	T1		270	94.5	81	94.5	0
	T2		270	94.5	81	47.25	47.25
	T3		270	94.5	81	0	94.5
Additional panicle fertilization treatments							
2020−2021	T4		317.25	94.5	81	94.5	47.25
	T5		364.5	94.5	81	94.5	94.5

### Soil alkali hydrolyzable N content

The soil was randomly sampled and pooled to form a composite of at least five spots from each plot at a depth of 0 to 20 cm using a 5-cm diameter soil auger. Following SR, the soil samples were collected at 10-day intervals, resulting in a total of nine samples. Soil samples were sieved to remove any plant residues and stones larger than 2 mm, then air-dried in a cool area before being subjected to determining the soil alkali hydrolyzable N by alkaline diffusion method (Zhang et al., 2022b).

### Nitrogen accumulation

A total of 20 rice plants (shoot parts) were sampled from each plot at five different stages, including PI, branch primordium differentiation (BPD), SPD, PMC, and pollen filling (PF). These plants were selected according to the average number of tillers in the respective treatment. The N content was determined through micro-Kjeldahl digestion and titration (Liu et al., 2022). The N accumulation was calculated as the product of N content and biomass at each growth stage. Differential accumulation of N was the difference between the N uptake at each stage, i.e., from PI to BPD, from BPD to SPD, from SPD to PMC, and from PMC to PF. The N accumulation per stem was calculated as the ratio of the aboveground N accumulation per unit area of rice to the number of tillers (or panicles at later stages).

### Extraction and quantification of BRs

At the SPD and PMC stages, 150 young panicles were collected from each plot to measure the concentration of BRs. The extraction and purification of BRs from the panicles were based on the method with modification (Zhang et al., 2020). Fresh panicles were frozen in liquid N, ground into a fine powder, transferred (0.5–0.8 g) to a 10-mL centrifuge tube, added 4 mL of acetonitrile for extraction, and refrigerated overnight at −20°C. Extraction, dehydration, and double layer-solid phase extraction (DL/SPE) were performed following the described method (Chen et al., 2009). The BRs were quantified using high-performance liquid chromatography-electrospray ionization-tandem mass spectrometry (HPLC-ESI-MS/MS). The levels of 24-epicastasterone (24-epiCS) and 28-homobrassinolide (28-homoBL) were measured, as they are the most important BRs in rice plants and exhibit high biological activity. Quantification (pmol g^−1^ fresh weight) was by the ratio of the total area converted based on the multiple reaction monitoring (MRM) of BRs, with a calibration by corresponding standard BRs. The Xcalibur data system (Thermo Fisher Scientific, Waltham, MA, USA) was used for data collection and analysis.

### Enumeration of spikelet differentiation and degeneration

One hundred plants, representing the average number of panicles of the plots, were sampled and measured at the heading stage. The numbers of surviving spikelets (NSS) and degenerated spikelets (NDeS) were recorded, with a white, significantly smaller spikelet on a rice panicle being regarded as a degenerated spikelet. The number of differentiated spikelets (NDiS) was estimated as the sum of NSS and NDeS, and the degeneration rate of spikelets (DRS) was determined as the ratio of NDeS to NDiS.

### Grain yield and yield components

The number of panicles per hill was estimated by averaging 60 randomly selected rice plants from each treatment. Twelve rice plants were sampled from each treatment to determine the number of spikelets per panicle, seed-setting rate, and 1,000-grain weight. The grain yield was determined by harvesting a 1-m² area at the center of each plot, with an adjustment of the grain moisture content to 14% (TDS-1G, Zhejiang Topu Yunnong Technology, Hangzhou, China).

### Data analysis

The experimental data underwent a three-way analysis of variance in IBM SPSS 25.0 (Chicago, IL, USA). Duncan’s multiple range test, with a *post-hoc* test at a 95% confidence interval, was used in comparison between the treatments. Path analysis was done by Pearson’s correlation. To establish a linear regression equation, the yield data underwent the Kolmogorov–Smirnov test for normality and a stepwise regression analysis on the yield and its component. Graphs were prepared in Origin 2022 (Origin Lab, Hampton, MA, USA).

## Results

### SR enhances grain yield consistently in *indica* and *japonica* rice, mainly via an increased spikelet number per panicle

**Figure 2:**
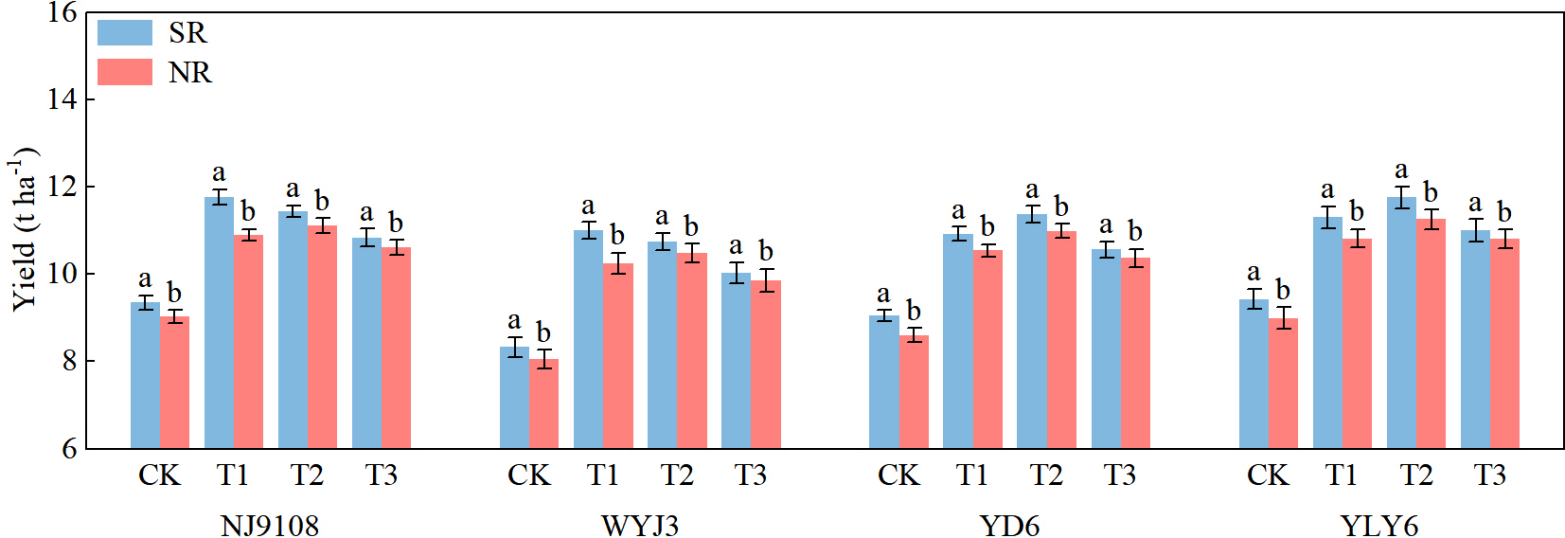
Effects of SR and panicle fertilization on the rice yields of *japonica* and *indica* in the past 3 years. SR, straw return; NR, no straw return; CK, no panicle fertilization; T1, panicle fertilization at the PI stage; T2, panicle fertilization at the PI and SPD stages; T3, panicle fertilization at the SPD stage; NJ9108, Nanjing 9108; WYJ3, Wuyunjing 3; YD6, Yangdao 6; YLY6, Yangliangyou 6. The vertical bars represent the mean ± standard error (*n* = 12). Different letters above the columns indicate significant differences between SR and NR for the same panicle fertilization treatment of the variety at the *p* = 0.05 level.

The results were consistent across the three experiment seasons (**Supplementary Table S1**); therefore, we present them as average values, except for several figures. Following SR, the average yields for *japonica* and *indica* rice increased by 4% and 3.69%, respectively, compared to NR practices (**Figure 2**). For SR, the highest yield for *japonica* rice was always observed under the T1 treatment, while it was in the T2 treatment under the NR. The highest yields for *indica* rice were consistently recorded under T2 treatment, irrespective of SR or NR. These indicate panicle fertilization split displayed different effects in *japonica* and *indica* rice.

Implementation of SR resulted in an insignificant reduction in the number of panicles (−2.02%); however, panicle fertilization treatments did not show a significant effect on the panicle number (**Table 2**). With SR, the spikelet number per panicle increased by 5.55%, with the maximums observed in T1 for *japonica* rice and T2 for *indica* rice. SR enhanced the seed-setting rate and 1,000-grain weight by 0.37% and 0.07%, respectively. In contrast, under NR conditions, the highest yield and number of spikelets per panicle for both *indica* and *japonica* rice were recorded under T2 treatment. The T3 treatment consistently produced the highest seed-setting rate and 1,000-grain weight for both *japonica* and *indica* rice, irrespective of SR and NR conditions.

**Table 2:** Effect of SR and panicle fertilization on yield components of *japonica* and *indica* rice in the past 3 years.

Straw treatment	Variety	Panicle fertilization treatment	Yield (t ha^−1^)	Panicles (×10^4^ ha^−1^)	Spikelets per panicle	Seed-setting rate (%)	Grain weight (g)
SR	NJ9108	CK	9.34 d	344.99 b	120.28 d	87.72 c	25.68 b
		T1	11.76 a	368.38 a	142.37 a	87.54 c	25.63 b
		T2	11.43 b	366.74 a	136.33 b	88.66 b	25.80 b
		T3	10.83 c	366.64 a	126.75 c	89.61 a	26.03 a
	WYJ3	CK	8.33 d	406.65 b	89.40 d	89.71 c	25.56 b
		T1	11.00 a	433.17 a	112.16 a	89.62 c	25.28 c
		T2	10.74 b	433.26 a	106.47 b	90.75 b	25.68 b
		T3	10.03 c	433.32 a	96.77 c	92.4 a	25.92 a
	YD6	CK	9.05 d	233.48 b	158.19 d	79.80 c	30.72 b
		T1	10.92 b	247.23 a	179.99 b	80.00 c	30.68 b
		T2	11.36 a	246.82 a	185.05 a	80.65 b	30.86 ab
		T3	10.56 c	246.67 a	169.06 c	81.71 a	31.01 a
	YLY6	CK	9.43 d	245.27 b	164.64 d	78.83 b	29.62 a
		T1	11.3 b	261.40 a	185.28 b	78.79 b	29.61 a
		T2	11.75 a	261.61 a	190.35 a	79.23 b	29.78 a
		T3	11.00 c	261.48 a	176.82 c	79.77 a	29.83 a
NR	NJ9108	CK	9.02 d	346.19 b	115.03 c	88.18 b	25.71 b
		T1	10.89 b	378.66 a	128.46 a	87.97 b	25.47 c
		T2	11.10 a	379.19 a	130.44 a	87.78 b	25.59 bc
		T3	10.61 c	379.40 a	121.27 b	88.73 a	26.01 a
	WYJ3	CK	8.05 d	412.34 b	83.90 d	90.03 b	25.86 a
		T1	10.24 b	450.94 a	100.02 b	89.12 c	25.51 b
		T2	10.48 a	451.40 a	102.47 a	89.07 c	25.45 b
		T3	9.86 c	451.78 a	92.61 c	90.86 a	25.94 a
	YD6	CK	8.60 d	236.17 b	150.72 d	79.52 b	30.40 c
		T1	10.53 b	249.72 a	172.2 b	79.79 b	30.71 b
		T2	10.98 a	250.03 a	177.97 a	80.01 b	30.86 ab
		T3	10.36 c	249.34 a	164.74 c	81.37 a	31.01 a
	YLY6	CK	8.99 c	248.09 b	155.63 d	79.10 b	29.45 c
		T1	10.81 b	264.33 a	175.21 b	79.01 b	29.56 bc
		T2	11.25 a	264.50 a	180.82 a	79.23 b	29.70 b
		T3	10.8 b	264.28 a	169.77 c	79.87 a	30.13 a
Source of variation							
Straw treatment (S)			–^**^	–^**^	–^**^	–^**^	ns
Variety (V)			–^**^	–^**^	–^**^	–^**^	–^**^
S × V			ns	ns	ns	ns	ns
Panicle fertilization treatment (P)			–^**^	–^**^	–^**^	–^**^	–^**^
S × P			–^**^	–^**^	–^**^	–^**^	–^*^
V × P			–^**^	–^**^	–^**^	–^**^	–^**^
S × V × P			ns	ns	–^**^	–^**^	–^**^

Different letters indicate statistically significant differences at the p = 0.05 level within the same variety of the same straw treatment.

ns, not significant at the p = 0.05 level.

^*^p = 0.05; ^**^p = 0.01—levels of significance.

Correlation and path analysis revealed a robust positive correlation between the number of spikelets per panicle and yield, with the former displaying the maximum direct path coefficient to the yield, indicating it was the most important contributor to yield change (**Table 3**).

**Table 3:** Correlation and path analysis of yield and its components.

Variety	Yield components	Correlation	Direct path coefficient	Indirect path coefficient			
				Panicles	Spikelets per panicle	Seed setting rate	1,000-grain weight
NJ9108	Panicles	0.652^**^	0.469		0.148	−0.12	−0.046
	Spikelets per panicle	0.862^**^	0.785	0.248		−0.13	−0.198
	Seed setting rate	0.051	0.195	−0.05	−0.032		0.134
	1,000-grain weight	0.045	0.155	−0.015	−0.039	0.106	
WYJ3	Panicles	0.602^**^	0.448		0.09	−0.021	−0.052
	Spikelets per panicle	0.877^**^	0.858	0.173		−0.147	−0.386
	Seed setting rate	0.029	0.131	−0.006	−0.022		0.081
	1,000-grain weight	−0.250^*^	0.107	−0.013	−0.048	0.066	
YD6	Panicles	0.792^**^	0.309		0.163	0.046	0.01
	Spikelets per panicle	0.893^**^	0.738	0.39		0.134	0.06
	Seed setting rate	0.290^**^	0.202	0.087	−0.021		0.041
	1,000-grain weight	−0.256^*^	0.145	0.005	0.012	−0.075	
YLY6	Panicles	0.805^**^	0.378		0.191	0.126	0.072
	Spikelets per panicle	0.897^**^	0.717	0.361		−0.089	0.04
	Seed setting rate	0.187	0.134	0.045	−0.017		0.019
	1,000-grain weight	0.242^*^	0.111	0.021	0.006	0.016	

^*^p = 0.05 and ^**^p = 0.01, levels of significance.

### SR triggers differential response of spikelet differentiation and degeneration in *indica* and *japonica* cultivars

**Figure 3:**
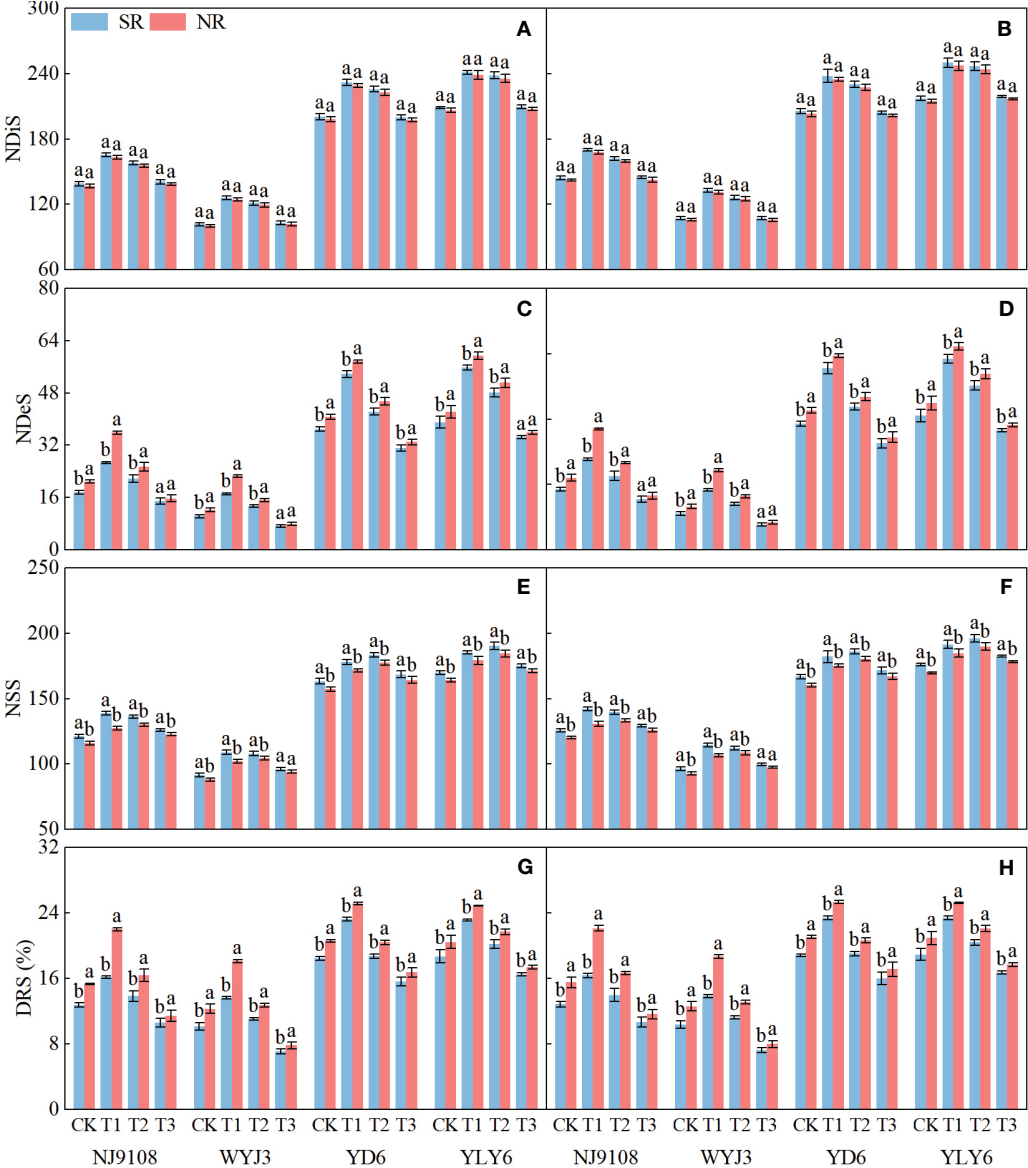
Effects of SR and panicle fertilization on rice spikelet differentiation and degeneration. NDiS, number of differentiated spikelets per panicle; NDeS, number of degenerated spikelets per panicle; NSS, number of surviving spikelets per panicle; DRS, degeneration rate of spikelets per panicle. **(A, C, E, G)** 2020; **(B, D, F, H)** 2021. Vertical bars represent the mean ± standard error (*n* = 4). Different letters above the columns indicate significant differences between SR and NR for the same panicle fertilization treatment at the *p* = 0.05 level.

In comparison with NR, SR did not significantly affect NDiS, but it significantly reduced NDeS and DRS (except T3) and consequently increased NSS (**Figure 3**). NDiS in *japonica* and *indica* rice exhibited subtle (insignificant) increments of 1.41% and 1.2%, respectively (**Figures 3A, B**). Under SR, the NDeS decreased by 17.46% and 6.61% (**Figures 3C, D**), the DRS reduced by 18.24% and 7.73% (**Figures 3G, H**), whereas the NSS increased by 4.72% and 3.3%, respectively, in *japonica* and *indica* rice (**Figures 3E, F**).

Irrespective of SR or not, N application at the PI stage significantly enhanced NDiS, with the maximum observed under T1 treatment in both *japonica* and *indica* rice (**Figures 3A, B**). In respective comparisons with T3 and T2 treatments, T1 increased NDiS by 4.64% and 20.24% in *japonica* rice and 2.16% and 15.41% in *indica* rice. On the other hand, N application later at the SPD stage significantly reduced NDeS and DRS, with the minimum values observed under T3 treatment for both *japonica* and *indica* rice. Compared to T1 and T2 treatments, NDeS declined by 55.68% and 46.9% in *japonica* rice and 40.16% and 30.95% in *indica* rice, respectively (**Figures 3C, D**); for DRS, the corresponding numbers were 40.83%, 32.19%, 27.42%, and17.99%, respectively (**Figures 3G, H**).

In terms of NSS, T1 treatment led to the highest NSS for *japonica* rice under SR implementation, increased by 10.3% and 1.69% in comparison to T3 and T2 treatments, respectively. The maximum NSS for *indica* rice was observed under T2 treatment, an increase of 8.42% and 2.61% relative to T3 and T1 treatments, respectively (**Figures 3E, F**). Under NR conditions, both *japonica* and *indica* rice achieved their highest NSS under T2 treatment, being 8.55% and 2.14% more than T3 treatment, and 7.51% and 3% more than T1 treatment, in *japonica* and *indica* rice, respectively.

In short, SR enhanced the number of spikelets, not via more spikelet differentiation but by reducing spikelet degeneration. This effect was magnified by panicle fertilization because panicle fertilization promoted the differentiation of spikelets. Panicle fertilization at a later stage (i.e., at the SPD stage) could not enhance spikelet differentiation and consequently did not remarkably promote the number of surviving spikelets.

### N accumulation per stem impacts spikelet differentiation and degeneration differentially in *indica* and *japonica* rice

**Figure 4:**
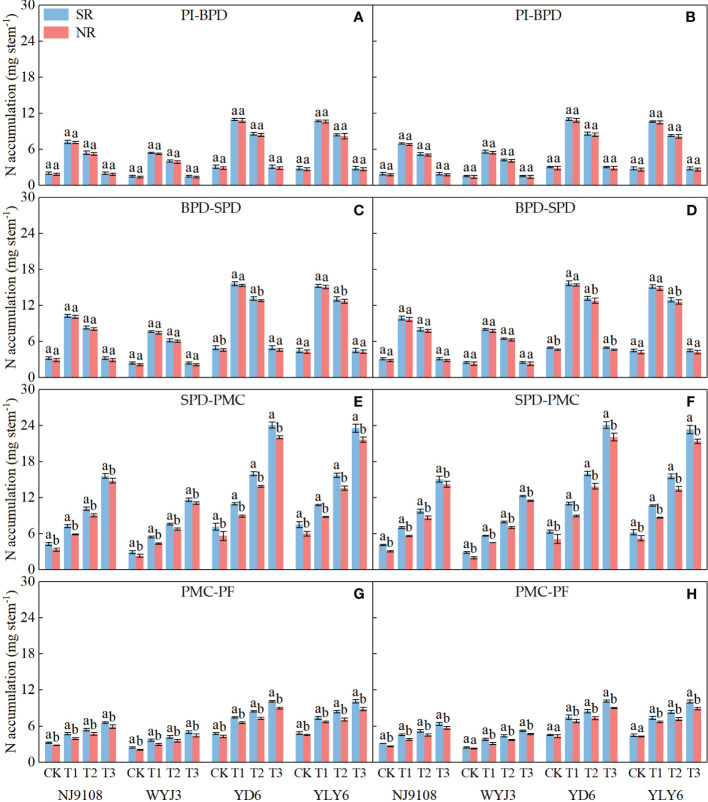
Effect of SR and panicle fertilization on N accumulation per stem during the panicle initiation to pollen filling stage. PI, panicle initiation; BPD, branch primordium differentiation; SPD, spikelet primordium differentiation; PMC, pollen mother cell meiosis; PF, pollen filling. **(A, C, E, G)** 2020; **(B, D, F, H)** 2021. Vertical bars represent the mean ± standard error (*n* = 4). Different letters above the columns indicate significant differences between SR and NR for the same panicle fertilization treatment at the *p* = 0.05 level.

Compared with NR, SR did not alter N accumulation per stem (NA) significantly during the PI-SPD stage (**Figures 4A–D**), but it did substantially increase NA during the SPD-PF stage (**Figures 4E–H**). Under SR, NA increased by 4.48% and 2.8% during the PI-BPD stage for *japonica* and *indica* rice, respectively (**Figures 4A, B**). Correspondingly, NA increased by 4.67% and 3.4% during the BPD-SPD stage (**Figures 4C, D**); by 13.62% and 14.96% during the SPD-PMC stage (**Figures 4E, F**); and by 16.06% and 12.55% during the PMC-BF stage for the two types of varieties, respectively (**Figures 4G, H**). Nitrogen application at the PI or SPD stages notably increased NA during the PI-SPD and SPD-PF stages, respectively. During the PI-SPD stage, the highest NA was observed under T1 treatment for both *japonica* and *indica* rice, followed by T2 treatment, with the lowest NA detected in T3 treatment. In contrast, during the SPD-PF stage, the highest NA was in the T3 treatment, followed by T2, with T1 being the lowest.

**Figure 5:**
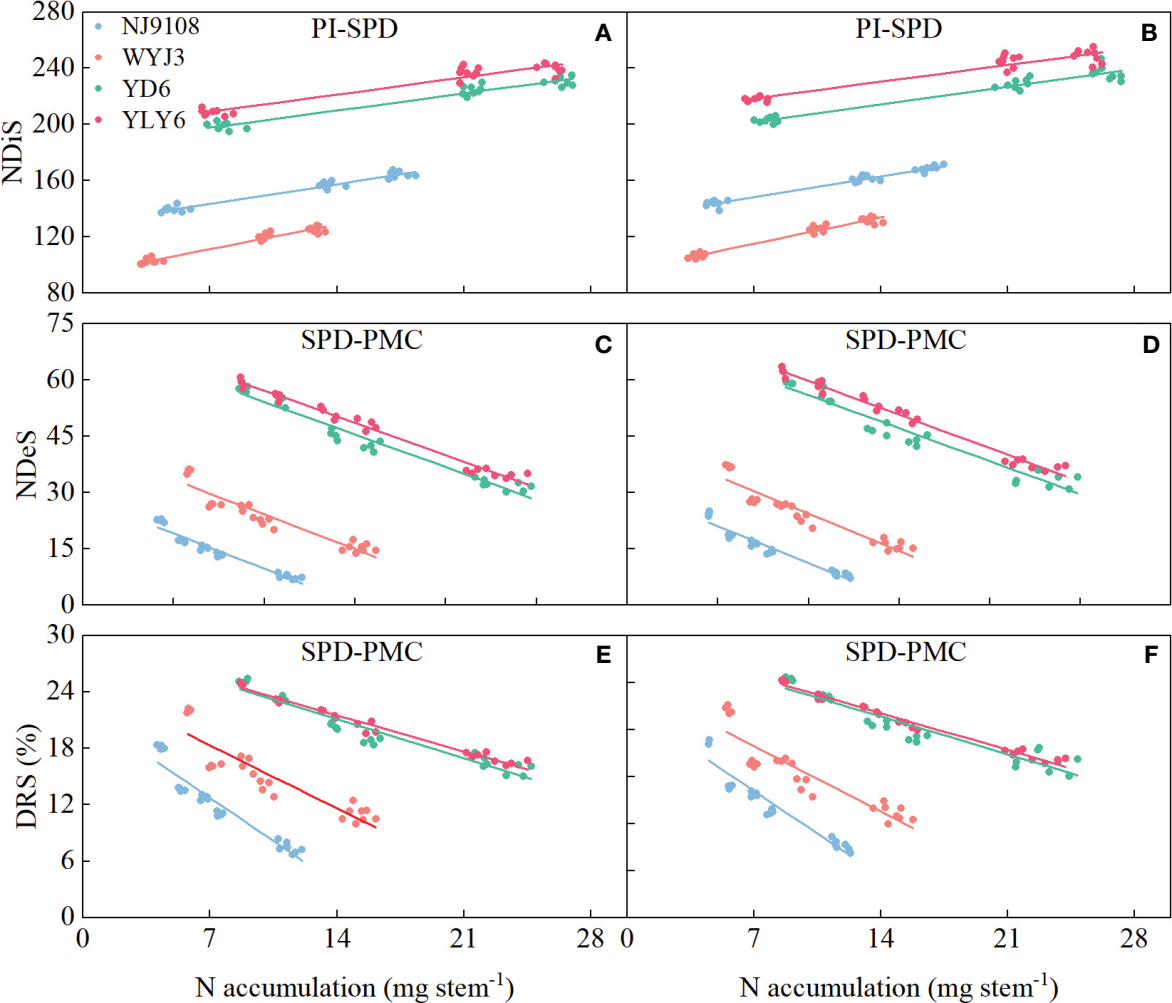
Correlation between differentiated spikelets (NDiS), degenerated spikelets (NDeS), and degeneration rate of spikelets (DRS) with N accumulation per stem during panicle initiation (PI)-spikelet primordium differentiation (SPD), and SPD-pollen mother cell meiosis (PMC) stages. **(A, C, E)** 2020; **(B, D, F)** 2021.

Path analysis demonstrated that NA during the BPD-SPD stage impacted most significantly on the NDiS and NSS, while NA during the PI-BPD stage contributed the greatest to the NDeS and DRS (**Table 4**). Significant correlations were found between the NDiS and NA during the PI-SPD stage (**Figures 5A, B**), with the linear model equation slope for *japonica* rice consistently greater than that of *indica* rice (**Supplementary Table S2**). Similarly, significant correlations were observed between NDeS, DRS, and NA during the SPD-PMC stage (**Figures 5C–F**). However, for *japonica* rice, the slope of the linear model equation was consistently smaller than that of *indica* rice (**Supplementary Table S2**), indicating that, for an equivalent increment of NA, it enhanced more spikelet differentiation and caused less spikelet degeneration in *japonica* rice than in *indica* rice.

**Table 4:** Path analysis of N accumulation in different phases leads to spikelet differentiation and degeneration.

Spikelets	Phases	Direct path coefficient			
		NJ9108	WYJ3	YD6	YLY6
NDiS	PI-BPD	0.128	0.233	−0.441	0.237
	BPD-SPD	0.788	0.651	1.267	0.74
	SPD-PMC	−0.226	−0.434	−0.463	0.162
	PMC-PF	0.248	0.487	0.438	−0.109
NDeS	PI-BPD	1.735	0.772	2.242	1.354
	BPD-SPD	−0.808	0.116	−1.498	−0.38
	SPD-PMC	0.525	0.125	−0.546	0.094
	PMC-PF	−0.839	−0.488	0.204	−0.356
NSS	PI-BPD	−1.362	−0.151	−3.055	−0.916
	BPD-SPD	1.967	0.876	3.721	1.681
	SPD-PMC	−0.828	−0.712	−0.243	0.193
	PMC-PF	1.145	1.021	0.548	0.158
DRS	PI-BPD	1.895	0.755	2.966	1.582
	BPD-SPD	−1.048	0.046	−2.313	−0.705
	SPD-PMC	0.652	0.202	−0.483	0.024
	PMC-PF	−1.074	−0.716	−0.003	−0.449

### BR levels increase along with the N accumulation at the SPD and PMC stages

**Figure 6:**
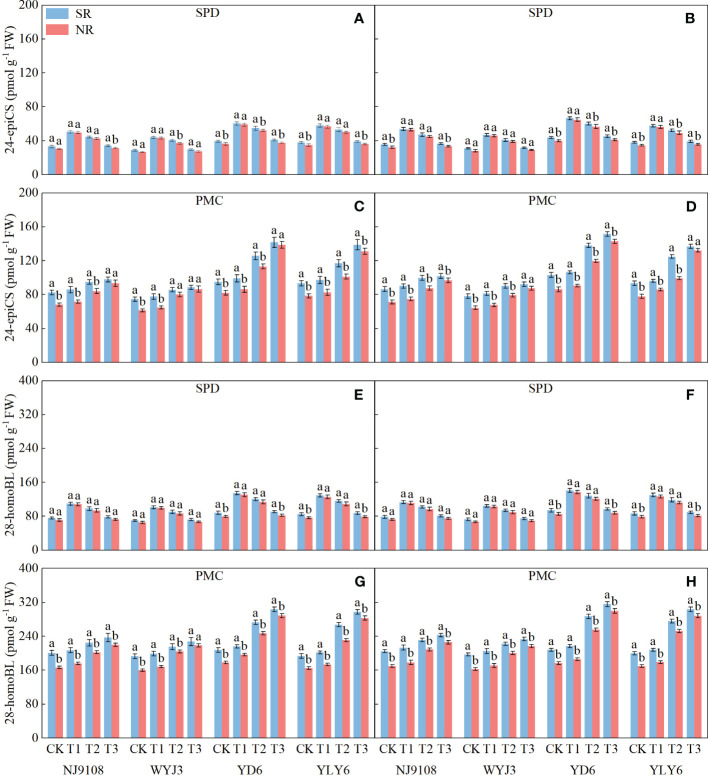
Effect of SR and panicle fertilization on the content of 24-epiCS and 28-homoBL in young rice panicles at spikelet primordium differentiation (SPD) and pollen mother cell meiosis (PMC) stages. **(A, C, E, G)** 2020; **(B, D, F, H)** 2021. Vertical bars represent the mean ± standard error (*n* = 4). Different letters above the columns indicate significant differences between SR and NR for the same panicle fertilization treatment at the *p* = 0.05 level.

In comparison to NR, SR had no significant effect on the levels of BRs (24-epiCS and 28-homoBL) in young panicles at the SPD stage (**Figures 6A, B, E, F**); however, at the PMC stage, BRs were significantly increased under SR (**Figures 6C, D, G, H**). The levels of 24-epiCS (**Figures 6A, B**) and 28-homoBL (**Figures 6E, F**) increased by 6% and 5.34%, respectively, in *japonica* rice and by 6.04% and 6.68% in *indica* rice at the SPD stage. At the PMC stage, their levels increased by 13.6% and 13.21% in *japonica* rice and by 12.73% and 11.32% in *indica* rice, respectively (**Figures 6C**, **D**, **G**, **H**).

Applying N at either the PI or SPD stage significantly affected the levels of BRs during panicle development. At the SPD stage, both *japonica* and *indica* rice showed the highest level of BRs under the T1 treatment, followed by the T2 treatment, while the lowest BR level was detected in the T3 treatment (**Figures 6A, B, E, F**). Conversely, at the PMC stage, the highest content of BRs was observed under T3 treatment and the lowest was detected under T1 treatment for both rice types (**Figures 6C, D, G, H**).

**Figure 7:**
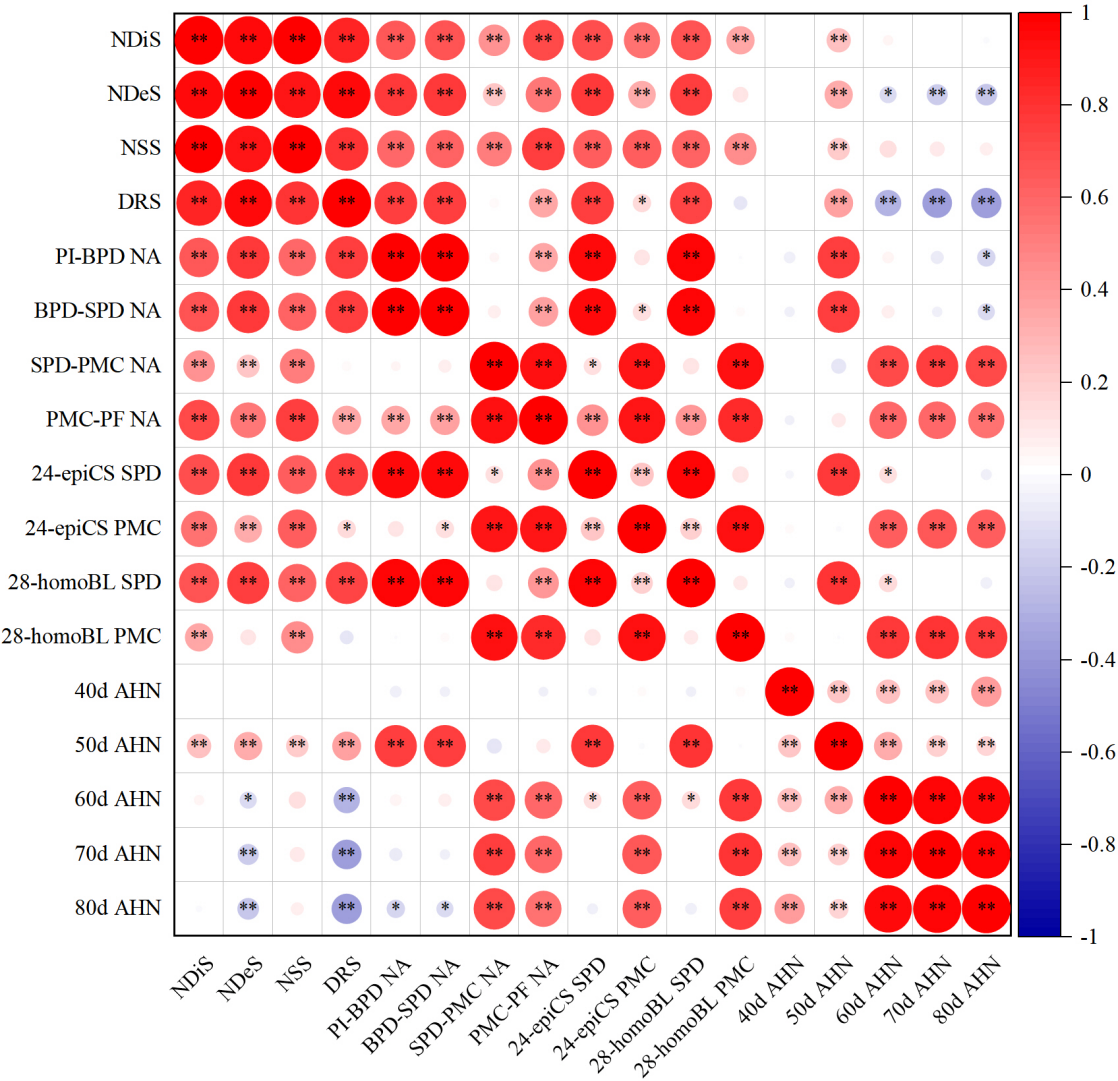
Correlations of spikelet differentiation and degeneration, N accumulation per stem (NA), level of brassinosteroids, and soil alkali hydrolyzable N. ^*^ *p* = 0.05 and ^**^ *p* = 0.01—levels of significance.

Additionally, the level of BRs at the SPD stage was significantly correlated with NA during the PI-SPD stage. Similarly, the level of BRs at the PMC stage was significantly correlated with NA during the SPD-PMC stage (**Figure 7**).

### Additional N application increment at the SPD stage verified the N supplement effect by SR

**Figure 8:**
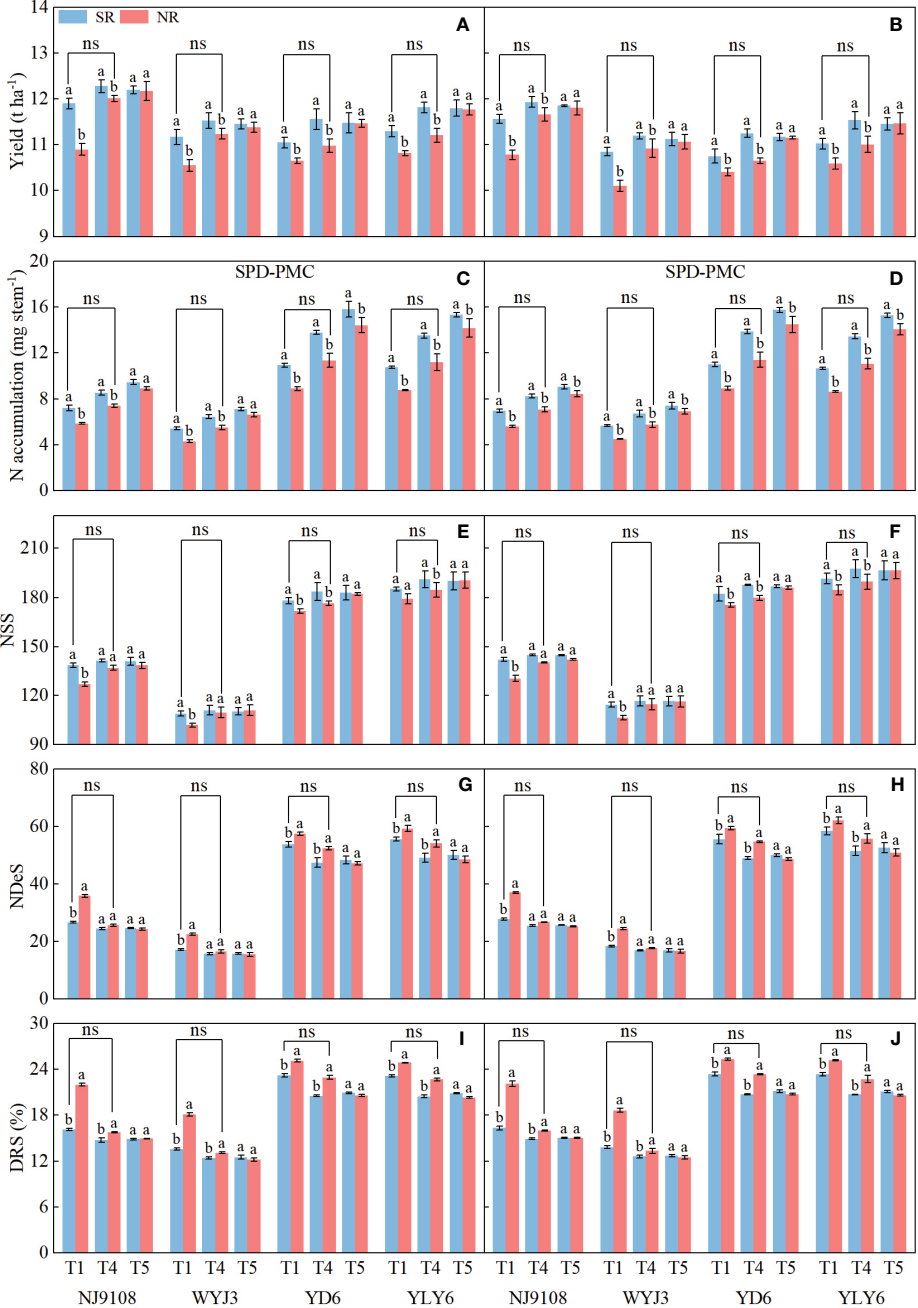
Effects of additional N fertilization at spikelet primordium differentiation stage (SPD) on yield, N accumulation per stem from SPD to pollen mother cell meiosis (PMC) stage, number of surviving spikelets (NSS), number of degenerated spikelets (NDeS), and degeneration rate of spikelets (DRS). **(A, C, E, G, I)** 2020; **(B, D, F, H, J)** 2021. Vertical bars represent the mean ± standard error (*n* = 4). Different letters above the columns indicate significant differences between SR and NR for the same panicle fertilization treatment at the *p* = 0.05 level.

To verify whether the impact of SR was mainly because of its N supplement effect, we introduced additional panicle fertilization rate treatments (T4 and T5) in 2020 and 2021 (**Table 1**). Following SR, increasing N application at the SPD stage indeed augmented the yield (**Figures 8A, B**) and NA during the SPD-PMC stage (**Figures 8C, D**), with a mild enhancement of NSS (**Figures 8E, F**) and a decrease of NDeS (**Figures 8G, H**) and DRS (**Figures 8I, J**) in both *japonica* and *indica* rice. The highest yield and NSS were obtained in T4, and the maximum NA was obtained in T5 treatment; the lowest NDeS and DRS were observed in T4 treatment. Under NR, increasing NA at the SPD stage significantly improved yield by increasing the NSS and substantially reducing NDeS and DRS in both *japonica* and *indica* rice. The highest yield, NA, and NSS were all observed in T5 treatment, and there appeared the lowest NDeS and DRS as well. Under NR, the yield, NA, NSS, NDeS, and DRS in both *japonica* and *indica* rice under T4 treatment exhibited no significant divergence from those under T1 treatment in SR, suggesting that SR promoted yield by more NSS mainly due to the contribution of N supplement during the SPD stage.

**Figure 9:**
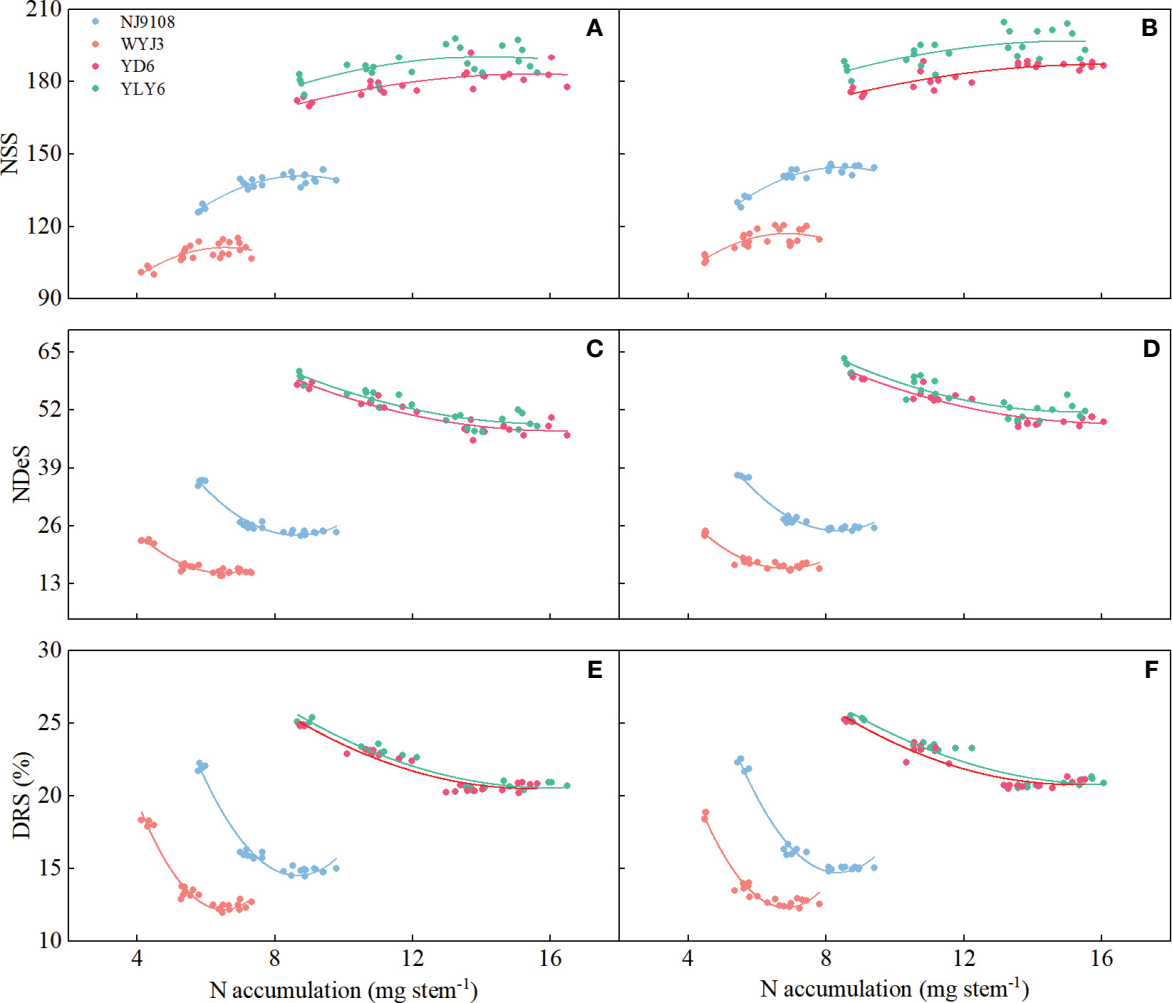
Correlation between number of surviving spikelets (NSS), number of degenerated spikelets (NDeS), and degeneration rate of spikelets (DRS) with N accumulation per stem at pollen mother cell meiosis stage. **(A, C, E)** 2020; **(B, D, F)** 2021.

In a polynomial regression of NA with NSS, NDeS, and DRS, differential responses were clearly noticeable in *japonica* and *indica* rice based on the pooled data from T4 and T5 treatments. Significant correlations were all found between NSS, NDeS, DRS, and NA, respectively, during the SPD-PMC stage (**Figures 9A–F**). The coefficient values of the second-degree component in the regression model were all negative for NSS and positive for NDeS, indicating that at a certain NA level, NSS could achieve a maximum and NDeS a minimum, respectively. It is interesting to note that the absolute coefficient values of the second degree were consistently greater in *japonica* rice (WYJ3 and NJ9108) than that of *indica* rice (YD6 and YLY6, **Supplementary Table S3**), indicating that the range of NA for reaching maximum NSS or minimum NDeS was way narrower in *japonica* rice than that in *indica* rice. Thus, handling panicle N fertilization to reach optimal NA for more NSS is a more delicate work in *japonica* than in *indica* rice.

NA is the product of N content and dry matter accumulation per stem. When correlating N content and dry matter accumulation per stem with NA (**Table 5**), it was clear that both factors exhibited a highly significant correlation with NA, with dry matter accumulation contributing more to NA in *japonica* rice and N content contributing more to NA in *indica* rice. This suggests that dry matter accumulation was more of a limiting factor for N uptake in *japonica*, while raising N content in *indica* rice probably would be more effective.

**Table 5:** Correlation and path analysis of N accumulation, dry matter, and N content of rice at the pollen mother cell meiosis stage.

Variety	Factors	Correlation	Direct path coefficient	Indirect path coefficient	
				Dry matter	N content
NJ9108	Dry matter	0.918^**^	0.903		0.038
	N content	0.396^**^	0.358	0.015	
WYJ3	Dry matter	0.927^**^	0.983		−0.172
	N content	0.150	0.322	−0.056	
YD6	Dry matter	0.683^**^	0.651		0.029
	N content	0.752^**^	0.723	0.032	
YLY6	Dry matter	0.660^**^	0.664		−0.006
	N content	0.738^**^	0.741	−0.004	

^*^p = 0.05 and ^**^p = 0.01—levels of significance.

### The fate of spikelet development is highly correlated with the level of soil alkali hydrolyzable N during the PI-SPD stage

**Figure 10:**
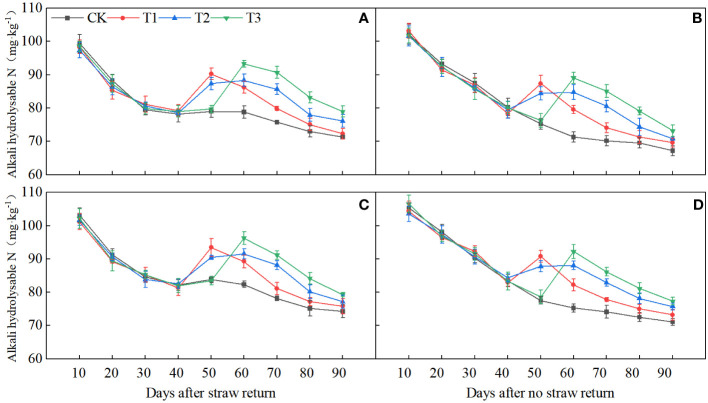
Dynamics of soil alkali hydrolyzable N from 0 to 90 days in SR and NR. **(A, C)** 2020, SR; **(B, D)** 2021, NR.

Direct monitoring of the N status in the soil revealed that the contents of alkali hydrolyzable N were very high at 10 days post-SR. Split top-dressing panicle fertilizers led to a significant increase in them at 50 and 60 days, respectively. In comparison to NR, the alkali hydrolyzable N content of the soil decreased by 4.99% between 0 and 40 days but increased by 5.15% between 50 and 90 days (**Figure 10**).

During the 40–50 days after SR, which coincided with the spikelet differentiation stage (**Supplementary Figure S1**), the alkali hydrolyzable N content of the soil showed significant positive correlations with NDiS, NDeS, NSS, DRS, and NA during the PI-SPD stage, as well as with the levels of BRs at the SPD stage (**Figure 7**). By contrast, during the 50–70 days after SR, which overlapped with the spikelet degeneration stage (**Supplementary Figure S1**), the alkali hydrolyzable N content of the soil showed significant negative correlations with NDeS and DRS, while significant positive correlations with NA during the SPD-PF stage and BR content at the PMC stage were observed.

## Discussion

### Yield

The effect of SR on rice growth largely depends on the amount and method of SR, soil fertility, and others (Liu et al., 2019; Qaswar et al., 2019; Sharma et al., 2020; Wang et al., 2020a; Wang et al., 2020b; Ali et al., 2021). Tiller occurrence in the early stage of rice growth is usually inhibited by SR due to its inhibitory effects of straw decomposition, toxic substance transit accumulation, and competition for nutrients, especially N from soil (Wang et al., 2019; Wang et al., 2020a; Li et al., 2023). As straw decomposes and releases nutrients, the inhibitory effect fades way to the promotion effect, which enhances rice growth in the middle and later stages (Ran et al., 2022; Zhang et al., 2022a; Liu et al., 2023). This dynamic in soil nutrients leads to a slowdown of tillering and eventually fewer panicles, but increases in spikelet number, seed-setting rate, and 1000-grain weight to varying degrees (Liu et al., 2019; Li et al., 2023). Our study also corroborated the tendency, as SR consistently enhanced the yield by an average of 3.77% over NR (**Figure 2**), with an average decrease of 2.64% in panicle number and an increase in the number of spikelets per panicle, seed-setting rate, and 1,000-grain weight by 5.66%, 0.54%, and 0.36%, respectively (**Table 2**). Path analysis proved that the increase in spikelet number per panicle was a major player in the yield enhancement.

The application of panicle fertilizer is crucial management in the high-yield cultivation of rice. Previous studies have indicated that panicle fertilization should be adjusted according to rice panicle size type (Zhang et al., 2013) or by the N utilization efficiency of the cultivar (Ju et al., 2021). The present experiments revealed significant differences in the optimal panicle fertilization pattern between *indica* and *japonica* rice following SR and NR installments (**Figure 2**). Under NR, both *indica* and *japonica* rice showed a similar response to panicle fertilization treatments. On the contrary, after SR, the yield of *japonica* rice was the highest under the T1 treatment, while that of *indica* rice was under the T2 treatment. These suggest that the optimal panicle fertilization split ratio should be different between *japonica* and *indica* rice. We hypothesize that the traditional strategy of equal split of panicle fertilizer could not supply enough N to *japonica* rice under SR conditions, so a higher proportion should tip toward the PI stage. Results from additional treatments of T4 and T5 supported this, but more solid evidence is needed. In general, these results indicate that customizing panicle fertilization strategies basing solely on the panicle size type of rice is insufficient, especially when SR is commonly adopted.

### Spikelet differentiation and degeneration

Among rice yield components, the number of spikelets per panicle is highly adjustable. Breeding for larger panicles, i.e., increasing the number of spikelets per panicle, has been the main approach to obtaining high grain yields (Sheehy et al., 2001; Vriet et al., 2012; González-Navarro et al., 2015). Though SR has been known to increase the spikelet number (Comino et al., 2010; Zhang et al., 2021; Zhang et al., 2022a; Li et al., 2023), few studies have explored the effect of SR on how it forms. The differentiation and degeneration of spikelets in rice are closely associated with the levels of BRs during panicle development. Nitrogen application regulates the biosynthesis and signal transduction of BRs in rice (Zhang et al., 2019). Our experiments revealed that SR increased the soil alkali hydrolyzable N during the rice panicle development period (**Figure 10**), enhanced the NA during the SPD-PF stage (**Figures 4E–H**), and lifted the BR levels in the young panicles at the PMC stage (**Figures 6C, D, G, H**). Consequently, NDiS increased marginally by 1.31%, but NDeS and DRS decreased significantly by 12.02% and 12.98%, respectively. In the end, NSS increased by 4.04% (**Figure 3**). This clearly demonstrates that SR increases NSS by decreasing NDeS.

However, the difference in response of the maximal NSS of *japonica* and *indica* rice to SR has not been examined in previous research. Although SR can reduce NDeS, the decrease in amplitude in the *japonica* rice was significantly greater than that in the *indica* rice in the T1 treatment (**Figures 3C, D**). The NSS was the highest in the T1 treatment in *japonica* but appeared in the T2 treatment in *indica* rice reflecting their diverse responses in the number of differentiated and degenerated spikelets in the two types of rice.

The quadratic regression between NSS, NDeS, DRS, and NA (**Figure 9**) revealed that *japonica* rice cultivars could only obtain the maximum NSS at a rather narrower range of NA than that of *indica* rice, which also produces the minimum NDeS and DRS. Correlation and path analysis revealed that the contribution of dry matter to NA was more important for *japonica* rice, whereas in *indica* rice the N concentration had more play (**Table 5**). This suggests that reducing spikelet degeneration by enhancing NA should count on increasing the dry matter accumulation per stem more in *japonica* rice, while in *indica* this should shift more toward raising the N concentration, especially during the PMC-PF stage which is highly correlated with NSS (**Table 4**).

Additional N fertilizer rate at the SPD stage to simulate the effect of nutrient release from wheat straw on rice plants (**Figure 8**) proved the hypothesis that SR promotes NSS and yield formation mainly due to extra N availability at the panicle formation period. Application of N fertilizer under NR increased rice yield, NSS, and NA during the SPD-PMC stage (**Figures 8A–F**), and reduced NDeS and DRS (**Figures 8G–J**). Moreover, the yield, NA, NDeS, NSS, and DRS in higher panicle fertilization rates in NR (T4 treatment) did not differ significantly from those in light panicle fertilization in T1 treatment of SR, basically validating our hypothesis.

## Conclusions

Our 3-year field experiments indicated that split panicle fertilization under SR should shift more toward the PI stage (T1) in *japonica* rice, while equal split was still optimal (T2) for *indica* rice. Wheat SR increased the soil alkali hydrolyzable N concentration 40 days after rice transplantation, promoting N accumulation per stem during the SPD-PF stage of rice growth. Furthermore, SR increased the level of young spikelet BRs at the PMC stage, leading to a reduction in NDeS and an increment in NSS. For *japonica* rice, increasing the single-stem dry matter accumulation during meiosis had a greater effect on reducing DRS than the N content, whereas for *indica* rice, it required an increase in both N content and single-stem dry matter accumulation to significantly reduce the DRS. These findings suggest that panicle fertilization can be simplified to one use for *japonica* under SR but not for *indica* rice. In conclusion, our study provides valuable insight into the benefits of SR and highlights the necessity of variety-dependent strategies in panicle fertilization.

## Data availability statement

The original contributions presented in the study are included in the article/**Supplementary Material**. Further inquiries can be directed to the corresponding authors.

## Author contributions

XS: Conceptualization, Data curation, Formal Analysis, Investigation, Methodology, Resources, Writing – original draft, Writing – review & editing. XZ: Conceptualization, Data curation, Formal Analysis, Writing – original draft, Writing – review & editing. SW: Investigation, Writing – original draft. TF: Investigation, Writing – original draft. ZD: Investigation, Writing – original draft. YY: Investigation, Writing – original draft. ZW: Investigation, Writing – original draft. SZ: Investigation, Writing – original draft. JX: Investigation, Writing – original draft. JZ: Funding acquisition, Methodology, Project administration, Resources, Writing – review & editing. JJ: Methodology, Project administration, Resources, Writing – review & editing. JH: Funding acquisition, Resources, Supervision, Writing – review & editing. YLY: Conceptualization, Data curation, Formal Analysis, Methodology, Supervision, Writing – review & editing. YW: Funding acquisition, Project administration, Resources, Writing – review & editing. GD: Conceptualization, Data curation, Formal Analysis, Funding acquisition, Methodology, Project administration, Resources, Supervision, Validation, Writing – review & editing.
