# Supplementary material for: Simplified panicle fertilization is applicable to japonica cultivars, but splits are preferred in indica rice for a higher paddy yield under wheat straw return
Source: Front Plant Sci. 2024 Jan 30;15:1273774. doi: 10.3389/fpls.2024.1273774 (PMC10861670; doi:10.3389/fpls.2024.1273774)
Supplement: Supplementary file 1 [file DataSheet_1.docx]

Supplementary Material

Simplified panicle fertilization applicable to japonica cultivars but splits preferred in indica rice for higher paddy yield under wheat straw return

Xiaowei Shu^1, #^, Xiaoxiang Zhang^2, #^, Shushen Wang^1^, Tong Fu^1^, Zhouyu Ding^1^, Ying Yang^1^, Zihan Wang^1^, Shiru Zhao^1^, Jiejiao Xu^1^, Juan Zhou^1^, Jing Ju^3^, Jianye Huang^1^, Youli Yao^1*^, Yulong Wang^1^, Guichun Dong^1*^

*** Correspondence:** Corresponding Author: yaoyl@yzu.edu.cn, gcdong@yzu.edu.cn

## Supplementary Tables

**TABLE 1** Effects of simplified panicle fertilizer application on yield and component factors of different rice varieties after SR.

| Year | Straw  Treatment | Variety | Panicle  fertilization  treatment | Yield  （t ha^-1^） | Panicles  (×10^4^ ha^-1^) | Spikelets  per panicle | Seed-setting  Rate (%) | Grain weight (g) |
| --- | --- | --- | --- | --- | --- | --- | --- | --- |
| 2019 | SR | NJ 9108 | 0:0 | 9.39 d | 357.11 b | 119.11 d | 86.12 b | 25.64 a |
|  |  |  | 2:0 | 11.81 a | 376.03 a | 144.42 a | 85.5 b | 25.46 a |
|  |  |  | 1:1 | 11.51 b | 375.84 a | 135.82 b | 87.9 a | 25.66 a |
|  |  |  | 0:2 | 10.89 c | 376.32 a | 126.49 c | 88.61 a | 25.81 a |
|  |  | WYJ 3 | 0:0 | 8.1 d | 408.16 b | 88.14 d | 89.69 c | 25.12 c |
|  |  |  | 2:0 | 10.98 a | 440.51 a | 109.73 a | 89.65 c | 25.34 bc |
|  |  |  | 1:1 | 10.68 b | 440.87 a | 104.02 b | 90.92 b | 25.61 ab |
|  |  |  | 0:2 | 9.89 c | 441.68 a | 93.54 c | 92.63 a | 25.84 a |
|  |  | YD 6 | 0:0 | 9 d | 234.51 b | 158.51 d | 79.24 b | 30.57 b |
|  |  |  | 2:0 | 10.96 b | 248.02 a | 182.91 b | 79.07 b | 30.55 b |
|  |  |  | 1:1 | 11.38 a | 247.53 a | 188.81 a | 79.86 b | 30.49 b |
|  |  |  | 0:2 | 10.49 c | 247.06 a | 169.88 c | 80.87 a | 30.92 a |
|  |  | YLY 6 | 0:0 | 9.62 d | 248.61 b | 164.85 d | 79.71 a | 29.46 a |
|  |  |  | 2:0 | 11.58 b | 264.04 a | 185.55 b | 79.97 a | 29.56 a |
|  |  |  | 1:1 | 12.02 a | 263.93 a | 191.9 a | 79.91 a | 29.72 a |
|  |  |  | 0:2 | 11.2 c | 264.08 a | 178.44 c | 80.5 a | 29.54 a |
|  | NR | NJ 9108 | 0:0 | 9.09 d | 357.79 b | 112.85 c | 87.7 a | 25.67 a |
|  |  |  | 2:0 | 10.99 b | 391.3 a | 125.89 a | 87.13 ab | 25.61 a |
|  |  |  | 1:1 | 11.23 a | 392.74 a | 129.2 a | 86.61 b | 25.54 a |
|  |  |  | 0:2 | 10.58 c | 393.13 a | 118.53 b | 87.73 a | 25.88 a |
|  |  | WYJ 3 | 0:0 | 7.87 d | 418.17 b | 81.31 c | 89.63 a | 25.83 a |
|  |  |  | 2:0 | 10.09 b | 459.95 a | 97.97 a | 88.31 b | 25.34 b |
|  |  |  | 1:1 | 10.4 a | 461.26 a | 101.04 a | 88.47 b | 25.23 b |
|  |  |  | 0:2 | 9.62 c | 461.26 a | 90.21 b | 90.01 a | 25.7 a |
|  |  | YD 6 | 0:0 | 8.56 d | 237.34 b | 149.52 d | 79.17 b | 30.48 a |
|  |  |  | 2:0 | 10.55 b | 251.08 a | 173.18 b | 78.73 b | 30.81 a |
|  |  |  | 1:1 | 10.97 a | 251.16 a | 180.08 a | 79.18 b | 30.66 a |
|  |  |  | 0:2 | 10.2 c | 250.85 a | 163.93 c | 80.61 a | 30.8 a |
|  |  | YLY 6 | 0:0 | 9.17 c | 251.38 b | 154.38 d | 80.48 a | 29.36 b |
|  |  |  | 2:0 | 11.03 b | 267.54 a | 175.87 b | 79.46 b | 29.51 b |
|  |  |  | 1:1 | 11.48 a | 267.14 a | 182.38 a | 79.89 ab | 29.49 b |
|  |  |  | 0:2 | 10.91 b | 267.61 a | 167.61 c | 80.67 a | 30.16 a |
| 2020 | SR | NJ 9108 | 0:0 | 9.41 d | 337.76 b | 119.62 d | 89.88 b | 25.92 b |
|  |  |  | 2:0 | 11.9 a | 364.79 a | 140.27 a | 89.37 b | 26.02 ab |
|  |  |  | 1:1 | 11.52 b | 360.45 a | 135.96 b | 90.22 b | 26.05 ab |
|  |  |  | 0:2 | 10.91 c | 360.63 a | 125.61 c | 91.34 a | 26.38 a |
|  |  | WYJ 3 | 0:0 | 8.49 d | 420.98 b | 86.97 d | 89.63 c | 25.89 a |
|  |  |  | 2:0 | 11.17 a | 443.92 a | 111.5 a | 89.46 c | 25.23 b |
|  |  |  | 1:1 | 10.92 b | 444.21 a | 105.49 b | 90.75 b | 25.69 a |
|  |  |  | 0:2 | 10.31 c | 443.76 a | 97.18 c | 92.43 a | 25.86 a |
|  |  | YD 6 | 0:0 | 9.17 d | 236.84 b | 156.29 d | 81.09 c | 30.57 a |
|  |  |  | 2:0 | 11.05 b | 250.5 a | 177.15 b | 81.18 c | 30.67 a |
|  |  |  | 1:1 | 11.5 a | 250.13 a | 181.95 a | 82.31 b | 30.71 a |
|  |  |  | 0:2 | 10.77 c | 250.5 a | 167.15 c | 83.6 a | 30.76 a |
|  |  | YLY 6 | 0:0 | 9.47 c | 247.7 b | 164.81 d | 78.27 b | 29.64 a |
|  |  |  | 2:0 | 11.29 b | 265.5 a | 182.9 b | 78.54 b | 29.61 a |
|  |  |  | 1:1 | 11.73 a | 265.13 a | 187.48 a | 78.88 ab | 29.92 a |
|  |  |  | 0:2 | 11.09 b | 265.5 a | 175.18 c | 79.62 a | 29.95 a |
|  | NR | NJ 9108 | 0:0 | 9.07 c | 339.07 b | 114.69 c | 89.78 ab | 25.98 ab |
|  |  |  | 2:0 | 10.9 b | 370.26 a | 128.48 a | 89.68 ab | 25.55 c |
|  |  |  | 1:1 | 11.15 a | 369.89 a | 130.49 a | 89.55 b | 25.8 bc |
|  |  |  | 0:2 | 10.72 b | 370.34 a | 121.57 b | 90.49 a | 26.3 a |
|  |  | WYJ 3 | 0:0 | 8.24 c | 422.82 b | 83.34 c | 90.09 b | 25.97 ab |
|  |  |  | 2:0 | 10.55 a | 462.17 a | 98.63 a | 90.07 b | 25.7 bc |
|  |  |  | 1:1 | 10.7 a | 461.5 a | 101.71 a | 89.53 b | 25.47 c |
|  |  |  | 0:2 | 10.17 b | 461.93 a | 91.85 b | 91.85 a | 26.1 a |
|  |  | YD 6 | 0:0 | 8.75 c | 239.75 b | 149.96 d | 80.87 b | 30.1 b |
|  |  |  | 2:0 | 10.65 b | 252.63 a | 169.17 b | 81.29 b | 30.67 a |
|  |  |  | 1:1 | 11.07 a | 252.88 a | 175.67 a | 80.96 b | 30.8 a |
|  |  |  | 0:2 | 10.58 b | 252.32 a | 163.1 c | 83.35 a | 30.85 a |
|  |  | YLY 6 | 0:0 | 9.02 c | 250.99 b | 156.84 c | 77.93 b | 29.41 b |
|  |  |  | 2:0 | 10.81 b | 268.19 a | 172.15 b | 79.32 a | 29.54 b |
|  |  |  | 1:1 | 11.2 a | 267.6 a | 177.44 a | 79.29 a | 29.77 b |
|  |  |  | 0:2 | 10.96 b | 267.7 a | 170.91 b | 79.35 a | 30.18 a |
| 2021 | SR | NJ 9108 | 0:0 | 9.22 d | 340.1 b | 122.11 d | 87.15 b | 25.48 b |
|  |  |  | 2:0 | 11.56 a | 364.31 a | 142.43 a | 87.75 b | 25.4 b |
|  |  |  | 1:1 | 11.27 b | 363.94 a | 137.21 b | 87.88 b | 25.7 ab |
|  |  |  | 0:2 | 10.7 c | 362.96 a | 128.15 c | 88.88 a | 25.89 a |
|  |  | WYJ 3 | 0:0 | 8.39 c | 390.79 b | 93.07 d | 89.81 c | 25.68 b |
|  |  |  | 2:0 | 10.85 a | 415.09 a | 115.25 a | 89.75 c | 25.27 c |
|  |  |  | 1:1 | 10.63 a | 414.71 a | 109.92 b | 90.57 b | 25.74 ab |
|  |  |  | 0:2 | 9.91 b | 414.53 a | 99.59 c | 92.14 a | 26.06 a |
|  |  | YD 6 | 0:0 | 8.97 d | 229.1 b | 159.76 d | 79.06 b | 31 b |
|  |  |  | 2:0 | 10.76 b | 243.19 a | 179.89 b | 79.75 b | 30.82 b |
|  |  |  | 1:1 | 11.22 a | 242.81 a | 184.39 a | 79.79 b | 31.39 a |
|  |  |  | 0:2 | 10.43 c | 242.44 a | 170.14 c | 80.65 a | 31.35 a |
|  |  | YLY 6 | 0:0 | 9.19 d | 239.5 b | 164.26 c | 78.5 ab | 29.77 a |
|  |  |  | 2:0 | 11.02 b | 254.68 a | 187.4 a | 77.86 b | 29.67 a |
|  |  |  | 1:1 | 11.49 a | 255.77 a | 191.66 a | 78.91 a | 29.71 a |
|  |  |  | 0:2 | 10.71 c | 254.86 a | 176.85 b | 79.18 a | 30.01 a |
|  | NR | NJ 9108 | 0:0 | 8.9 c | 341.7 b | 117.56 c | 87.05 b | 25.47 b |
|  |  |  | 2:0 | 10.78 ab | 374.42 a | 131.02 a | 87.1 b | 25.24 b |
|  |  |  | 1:1 | 10.94 a | 374.94 a | 131.63 a | 87.17 b | 25.43 b |
|  |  |  | 0:2 | 10.54 b | 374.74 a | 123.71 b | 87.97 a | 25.85 a |
|  |  | WYJ 3 | 0:0 | 8.03 c | 396.03 b | 87.04 c | 90.39 a | 25.78 ab |
|  |  |  | 2:0 | 10.1 a | 430.68 a | 103.46 a | 88.97 b | 25.48 b |
|  |  |  | 1:1 | 10.34 a | 431.45 a | 104.66 a | 89.22 b | 25.66 b |
|  |  |  | 0:2 | 9.77 b | 432.15 a | 95.77 b | 90.73 a | 26.03 a |
|  |  | YD 6 | 0:0 | 8.49 c | 231.44 b | 152.68 c | 78.53 c | 30.61 b |
|  |  |  | 2:0 | 10.4 b | 245.44 a | 174.24 a | 79.36 b | 30.66 b |
|  |  |  | 1:1 | 10.9 a | 246.02 a | 178.17 a | 79.88 ab | 31.12 a |
|  |  |  | 0:2 | 10.3 b | 244.85 a | 167.19 b | 80.17 a | 31.37 a |
|  |  | YLY 6 | 0:0 | 8.79 c | 241.89 b | 155.68 d | 78.9 ab | 29.59 b |
|  |  |  | 2:0 | 10.59 b | 257.26 a | 177.6 b | 78.23 b | 29.63 b |
|  |  |  | 1:1 | 11.06 a | 258.77 a | 182.65 a | 78.49 b | 29.83 ab |
|  |  |  | 0:2 | 10.52 b | 257.52 a | 170.8 c | 79.6 a | 30.05 a |

Different letters indicate statistically significant difference at *p* = 0.05 level within the same variety of the same straw treatment. ns: not significant at *p* = 0.05 level. * and **: significant at *p* = 0.05 and *p* = 0.01 level, respectively.

**TABLE 2** Relationships between the number of differentiated, degenerated, and surviving spikelets with N accumulation per stem.

| Year | Variety | NA_PI-SPD_ and NDiS | | NA_SPD-PMC_ and NDeS | | NA_SPD-PMC_ and DRS | |
| --- | --- | --- | --- | --- | --- | --- | --- |
|  |  | Equation | *R^2^* | Equation | *R^2^* | Equation | *R^2^* |
| 2020 | NJ9108 | y=1.98x+129.77 | 0.955 | y=-1.85x+44.71 | 0.884 | y=-0.95x+24.99 | 0.814 |
|  | WYJ3 | y=2.52x+93.69 | 0.952 | y=-1.88x+28.58 | 0.946 | y=-1.31x+21.93 | 0.915 |
|  | YD6 | y=1.74x+185.44 | 0.954 | y=-1.74x+71.67 | 0.966 | y=-0.59x+29.42 | 0.929 |
|  | YLY6 | y= 1.75x+196.78 | 0.919 | y=-1.72x+74.38 | 0.982 | y=-0.55x+29.22 | 0.978 |
| 2021 | NJ9108 | y=1.98x+129.77 | 0.967 | y=-1.97x+44.16 | 0.896 | y=-0.99x+25.17 | 0.829 |
|  | WYJ3 | y=2.68x+96.45 | 0.962 | y=-1.97x+30.89 | 0.941 | y=-1.29x+22.49 | 0.909 |
|  | YD6 | y=1.79x+185.44 | 0.935 | y=-1.76x+73.55 | 0.946 | y=-0.57x+29.42 | 0.900 |
|  | YLY6 | y=1.73x+206.18 | 0.903 | y=-1.79x+77.65 | 0.982 | y=-0.56x+29.55 | 0.981 |

NA_PI-SPD_: N accumulation per stem during the PI-SPD stages；NA_SPD-PMC_: N accumulation per stem during the SPD-PMC stages.

**TABLE 3** Quadratic model equations between number of differentiated, degenerated spikelets, and N accumulation per stem.

| Year | Variety | NSS | | NDeS | | DRS | |
| --- | --- | --- | --- | --- | --- | --- | --- |
|  |  | Equation | *R^2^* | Equation | *R^2^* | Equation | *R^2^* |
| 2020 | NJ9108 | y=-1.56x^2^+27.38x+20.67 | 0.835 | y=1.50x^2^-25.91x+135.39 | 0.956 | y=0.93x^2^-16.13x+84.23 | 0.971 |
|  | WYJ3 | y=-1.82x^2^+23.83x+33.03 | 0.628 | y=1.43x^2^-18.55x+75.72 | 0.922 | y=1.19x^2^-15.48x+62.54 | 0.958 |
|  | YD6 | y=-0.27x^2^+8.23x+119.14 | 0.608 | y=0.20x^2^-6.48x+99.78 | 0.885 | y=0.09x^2^-2.99x+44.52 | 0.947 |
|  | YLY6 | y=-0.38x^2^+10.89x+112.84 | 0.441 | y=0.21x^2^-6.58x+101.68 | 0.861 | y=0.10x^2^-3.13x+44.70 | 0.936 |
| 2021 | NJ9108 | y=-1.65x^2^+27.87x+26.39 | 0.901 | y=1.59x^2^-26.39x+134.65 | 0.966 | y=0.956x^2^-15.95x+81.17 | 0.829 |
|  | WYJ3 | y=-1.85x^2^+25.39x+29.74 | 0.657 | y=1.42x^2^-19.34x+82.47 | 0.926 | y=1.12x^2^-15.38x+64.86 | 0.961 |
|  | YD6 | y=-0.23x^2^+7.36x+127.89 | 0.717 | y=0.20x^2^-6.59x+102.57 | 0.895 | y=0.09x^2^-2.88x+44.11 | 0.924 |
|  | YLY6 | y=-0.29x^2^+8.89x+129.62 | 0.451 | y=0.25x^2^-7.71x+110.23 | 0.819 | y=0.11x^2^-3.22x+45.24 | 0.928 |

NA: N accumulation per stem

## Supplementary Figures


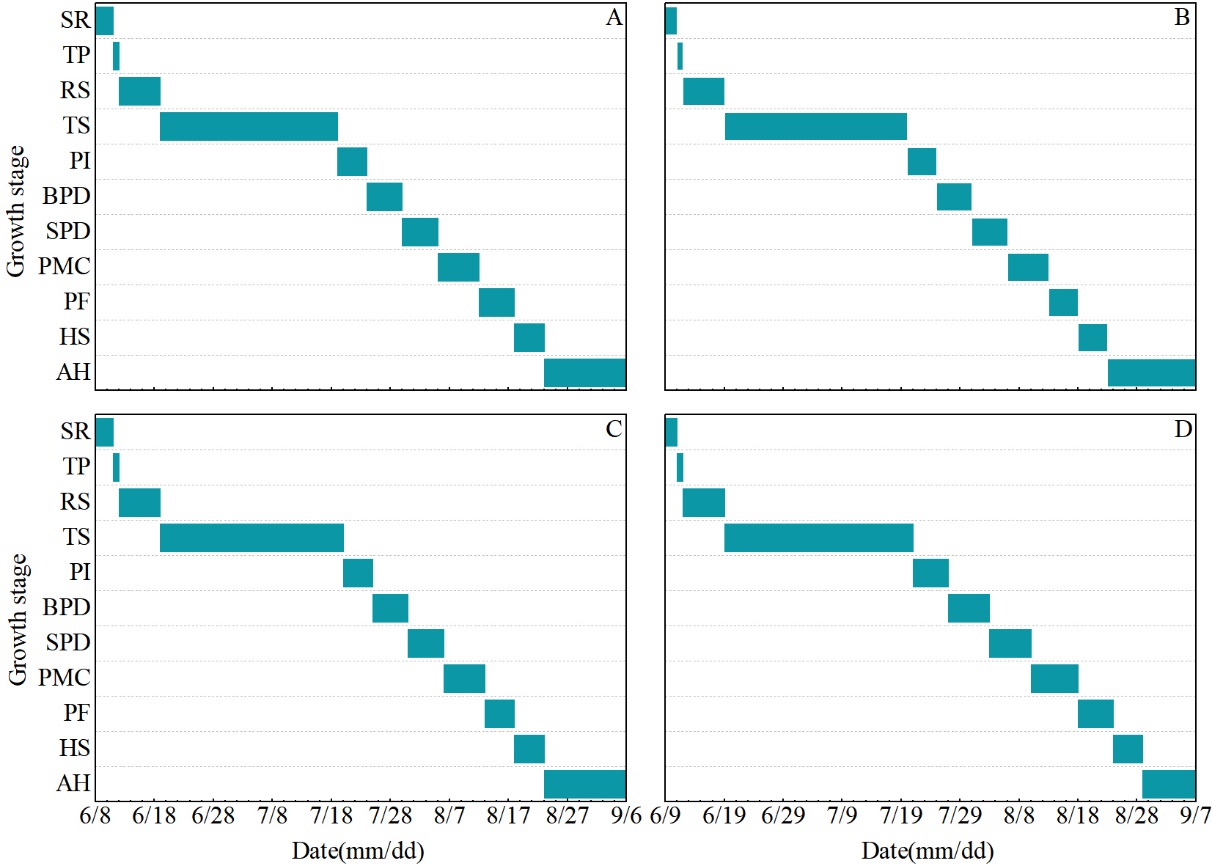


**FIGURE 1** Rice growth stage after SR (0-90d). A: *japonica* rice, 2020; B: *japonica* rice, 2021; C: *indica* rice, 2020; D: *indica* rice, 2021; SR: wheat straw return; TP: transplantation period; TS: tillering stage; PI: panicle initiation stage; BPD: branch primordium differentiation stage; SPD: spikelet primordium differentiation stage; PMC: pollen mother cell meiosis stage; PF: pollen filling stage; HS: heading stage; AH: after heading.

Our research focuses on the optimal panicle fertilization strategy for achieving high yield in indica and japonica rice after wheat straw return, which has direct relevance to the areas covered by the journal. The selected journal, frontiers in plant science, has consistently published high-quality articles in the field of crop and product physiology. Our manuscript contributes to the literature by analyzing the reasons for the differences in response of yield of indica and japonica rice to simplified panicle fertilizer after wheat straw return. Furthermore, our findings provide a valuable insight into the benefits of wheat straw return and highlights the necessity of varietal differential strategies in panicle fertilization. Given the extensive track record of the journal in publishing cutting-edge research within our field, we believe that our manuscript will not only fit within the journal's scope but also contribute to its ongoing intellectual discourse.
